# Clinical Setting Influences Off-Label and Unlicensed Prescribing in a Paediatric Teaching Hospital

**DOI:** 10.1371/journal.pone.0120630

**Published:** 2015-03-10

**Authors:** Petra Czarniak, Lewis Bint, Laurent Favié, Richard Parsons, Jeff Hughes, Bruce Sunderland

**Affiliations:** 1 Curtin University, Bentley, Western Australia, Australia; 2 Princess Margaret Hospital, Roberts Rd, Subiaco, Western Australia, Australia; 3 Department of Pharmaceutical Sciences, Utrecht University, The Netherlands; Centre Hospitalier Universitaire Vaudois, FRANCE

## Abstract

**Purpose:**

To estimate the prevalence of off-label and unlicensed prescribing during 2008 at a major paediatric teaching hospital in Western Australia.

**Methods:**

A 12-month retrospective study was conducted at Princess Margaret Hospital using medication chart records randomly selected from 145,550 patient encounters from the Emergency Department, Inpatient Wards and Outpatient Clinics. Patient and prescribing data were collected. Drugs were classified as off-label or unlicensed based on Australian registration data. A hierarchical system of age, indication, route of administration and dosage was used. Drugs were classified according to the Anatomical Therapeutic Chemical Code.

**Results:**

A total of 1,037 paediatric patients were selected where 2,654 prescriptions for 330 different drugs were prescribed to 699 patients (67.4%). Most off-label drugs (n = 295; 43.3%) were from the nervous system; a majority of unlicensed drugs were systemic hormonal preparations excluding sex hormones (n = 22, 32.4%). Inpatients were prescribed more off-label drugs than outpatients or Emergency Department patients (p < 0.0001). Most off-label prescribing occurred in infants and children (31.7% and 35.9% respectively) and the highest percentage of unlicensed prescribing (7.2%) occurred in infants (p < 0.0001). There were 25.7% of off-label and 2.6% of unlicensed medications prescribed across all three settings. Common reasons for off-label prescribing were dosage (47.4%) and age (43.2%).

**Conclusion:**

This study confirmed off-label and unlicensed use of drugs remains common. Further, that prevalence of both is influenced by the clinical setting, which has implications in regards to medication misadventure, and the need to have systems in place to minimise medication errors. Further, there remains a need for changes in the regulatory system in Australia to ensure that manufacturers incorporate, as it becomes available, evidence regarding efficacy and safety of their drugs in children in the official product information.

## Introduction

In Australia, it is a requirement that drugs to be marketed are licensed by the Therapeutics Goods Administration (TGA) to ensure they meet requirements for efficacy, safety and quality [1]. Many medications prescribed for children have not been evaluated in this population and dosages are often extrapolated from data obtained from adult trials. Developmental changes in children that occur with age, including gastric acidity, the activity of hepatic drug metabolising enzymes, renal function and drug receptor expression, influence the pharmacokinetic and pharmacodynamic effects of drugs [2]. These factors can render data extrapolated from clinical studies in adults inappropriate in children.

Off-label and unlicensed prescribing is not illegal in Australia and is often clinically appropriate. Reasons for off-label prescribing include administration to children outside the age range for which the product is licensed, the use of doses other than those stated in the approved Product Information, the use of alternative routes of administration or use for indications not approved in the license [3]. Reformulation of a registered drug (to obtain a desired dose or make the drug easier to administer), formulations manufactured under a special license, the use of unregistered drugs or the use of a non-pharmacological substance as a medicine are examples of unlicensed prescribing [3]. Unlicensed and off-label prescribing is a global phenomenon.

Several retrospective and prospective studies have been conducted in the United Kingdom (UK) [3–5], France [6], the Netherlands [7], Germany [8, 9], Switzerland [10], Finland [11, 12], Croatia [13], Italy [14], Portugal [15], across Europe [16], United States (US) [17], Brazil [18], Ireland [19], Israel [20], Malaysia [21] and Australia [1, 22, 23] in various hospital settings that have included Emergency Department patients, outpatients or inpatients (general and medical wards and paediatric and neonatal intensive care units). Most of the studies have been conducted over a period of weeks or months. The extent of off-label and unlicensed prescribing in paediatrics has been reported to range from 7 to 60%, with 28 to 100% of paediatric patients receiving at least one off-label or unlicensed drug [2, 7, 10, 11, 15, 16, 21]. The proportion of children that receive unlicensed or off-label drugs in the hospital setting has been shown to be higher in intensive care units and in those with complex diseases [4, 5, 16, 24].

Data on the extent of off-label and unlicensed prescribing in paediatrics in Australia are limited. A 10 week study that investigated the use of unlicensed and off-label drugs in a neonatal intensive care unit at the Royal Women’s Hospital, Melbourne reported its prevalence as 11% and 47% respectively, with 80% of patients receiving at least one off-label or unlicensed drug [1]. A retrospective study on the use of off-label medicines on a general paediatric ward at the Royal Hobart Hospital, Tasmania reported that 31.8% of medicines were used off-label and 57.3% of children received an off-label medicine [23]. In Western Australia, a study conducted in 1997 in one surgical ward and one medical ward at Princess Margaret Hospital reported a higher incidence (17%) of off-label and unlicensed prescribing on the medical ward but found that more children on the surgical ward (39%) received an off-label or unlicensed drug [22].

To improve evidence-based prescribing for children in Australia, a Paediatric Medicines Advisory Group was formed in 2007 to provide advice to the Therapeutics Goods Administration and Government on paediatric medicine issues. A Paediatric Medicines Dosing Resource, the AMH Children's Dosing Companion, an initiative of the Australian Health Ministers’ Advisory Council, became available in Australia in mid-2013 and provides detailed evidence-based and peer-reviewed dosing information with monographs on 230 drugs, including information on off-label uses [25]. Despite this, studies of off-label and unlicensed drug use in Australia are limited and have only investigated specific areas of paediatric hospital treatment. This study aimed to estimate the prevalence of off-label and unlicensed prescribing from an overall hospital perspective by randomly selecting patient records from all hospital settings (emergency, inpatient and outpatient) over a single year of hospital patients in a major paediatric teaching hospital in Western Australia.

## Method

A retrospective study was conducted at Princess Margaret Hospital. Medication chart records were randomly selected from a total population of 145,550 patient encounters of which 55,591 were from Emergency Department attendances, 24,425 from inpatients and 65,534 from outpatient admissions. The proportion of Emergency Department patients: inpatients: outpatients was maintained to generate a list of 1200 randomly selected cases. The justification for selecting this number of records for review was so that the 95% confidence intervals (CI) for the prevalence estimates would be no wider than ±3% (a measure of precision). Of the 1200 records, data from 1038 medication chart records from a single year (2008) were available and analysed for prescribing in the paediatric emergency, outpatient and inpatient departments. Each record had a unique identifying number, the code for which was lodged with the Chief Pharmacist at Princess Margaret Hospital. As patient medication records were de-identified prior to analysis, written informed consent from participants was not required for the study.

An event date in 2008 was recorded for each admission record, as well as any prescribing details (date of prescription, dosage form, dose, strength of drug, frequency of dosing), diagnosis, and relevant patient data including sex, date of birth, weight, height, past medical history, ceased medications and reasons for ceasing. If the event date was directly connected to another event in 2008 (on-going care), drugs prescribed for that event were also recorded. Patient records were accessed via the Patient Information Management Services and viewed in the Patient Information Management Services survey room at Princess Margaret Hospital.

The patient's age was classified according to the European Medicines Agency [26] age classification: newborns (zero to 27 days), infants and toddlers (28 days to 23 months), children (two to 11 years) and adolescents (12 to 18 years).

Following data collection, all prescribed drugs were classified as licensed, off-label or unlicensed according to the approved Product Information as shown in the 2008 edition of Monthly Index of Medical Specialities [27] that was current at the time of prescribing or the Therapeutic Goods Administration Product Information [28] data. Categories of off-label prescribing were defined sequentially as follows:

1. Age/ weight—administration of a prescription drug outside the age range or weight for which the product is licensed.
2. Indication—the use was for indications not described in the Product Information.
3. Route of administration—the use of alternative routes of administration other than the approved route for that formulation in the Product Information.
4. Dosage including dose frequency—the use of doses or dose frequencies other than those stated in the approved Product Information.

A prescription drug was considered off-label if it met at least one of the above four criteria according to the Therapeutic Goods Administration [28] or Monthly Index of Medical Specialities [27] Product Information.

A hierarchical approach adopted by Hsien et al [9] was used for this study on the basis that an off-label prescribed drug was defined by any one classification. In keeping with Hsien et al [9] all prescriptions were initially analysed for age so that drugs with no paediatric information or those prescribed for an age group for which the drug was not licensed were classified as off-label for age. The next level was indication, then route of administration and finally dosage (which included frequency of administration). Once drugs were classified into a category eg age, they were then not considered for possible classification into any subsequent category. Where the Therapeutic Goods Administration or Monthly Index of Medical Specialities Product Information provided a drug dose range (eg Painstop 1–2 yrs [10–12kg]: 5–6 mL), dosages administered outside this dose range were considered off-label. However, where the dose provided in the Therapeutic Goods Administration or Monthly Index of Medical Specialities Product Information was prescribed on a weight basis eg paracetamol 15mg/ kg/ dose, a variation of ± 10% was accepted to allow for practical dosage volumes [27, 28].

Drugs were classified as unlicensed if they were an unregistered drug, an unlicensed formulation of a registered drug or the use of a non-pharmacological substance as a medicine. Where a drug was classified as both off-label and unlicensed, the final classification was off-label. For any drugs where there was an uncertainty as to whether or not it was off-label or unlicensed, a conservative approach was adopted and it was classified as licensed. Prescribed drugs were coded based on the Anatomical Therapeutic Chemical classification.

Prescriptions for oxygen, parenteral nutrition and drugs used in research studies, were not included.

Simple descriptive statistics (means and standard deviations for continuous variables, frequencies and percentages for categorical variables) were determined for patients and prescription drugs. The Pearson Chi-square test was used to determine whether the proportion of patients receiving licensed, unlicensed and off-label drugs differed significantly between hospital settings. A similar analysis was used to compare proportions of drugs prescribed between the settings. A logistic regression model was used to compare the proportions of licensed drugs between different drug classes. Continuous variables were compared between settings using an Analysis of Variance. A p-value of less than 0.05 was taken to indicate a statistically significant association.

This study was approved by the Princess Margaret Hospital Ethics Committee (Audit 103QP) and the Human Research Ethics Committee at Curtin University (approval number PH-13–11).

## Results

**Fig 1:**
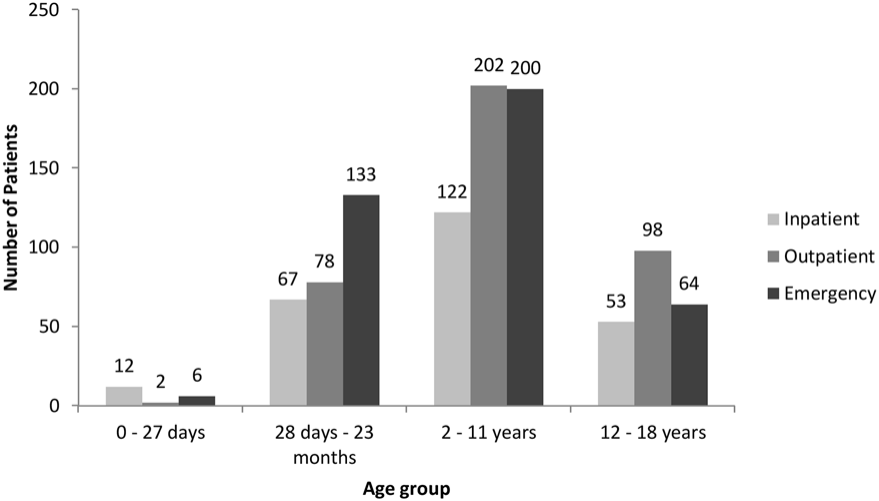
Age distribution of inpatients, outpatients and emergency department patients.

Data were collected from a total of 1038 medication chart records which were randomly selected from inpatients, outpatients and Emergency Department admissions. Of these, 59% were male and 41% were female. One patient, a 19 year old, was excluded from the study due to age. Most records (n = 403; 39%) were from the Emergency Department; 37% were outpatients (n = 380) and 24% inpatients (n = 254) and in each setting, a majority was male (58% in the Emergency Department, 55% outpatients, 65% inpatients). The average age (±standard deviation) of inpatients was 6.4 ± 5.4 years, outpatients 7.8 ± 5.2 years and Emergency Department patients 5.6 ± 4.8 years; with a median age in each setting of 5.4 years, 7.8 years and 4.0 years respectively (p = 0.0003). Fig. 1 shows that most patients were in the European Medicines Agency 2 to 11 year age group, with the Emergency Department having the most frequent attendance for the 28 days to 23 months age group.

Although the gender differences and frequencies of patients prescribed no drugs, licensed, off-label or unlicensed drugs was statistically significant (p = 0.0198) (Table 1), there was no statistical difference between genders in the proportions of cases prescribed no drugs (32.6% overall) or licensed drugs (31.0% overall, p = 0.1520). However, amongst the remaining 378 cases who were prescribed at least one off-label or unlicensed drug, 85.9% of the 142 females were prescribed an off-label drug compared with the 93.6% of the 236 males (p = 0.0121).

**Table 1:** Gender distribution of the number of patients prescribed no drugs, licensed drugs and off-label or unlicensed drugs.

Gender	No Drugs		Licensed Drugs		Off-label Drugs		Unlicensed Drugs	
	(n = 338)		(n = 321)		(n = 343)		(n = 35)	
**Male**								
(n = 607) (%)	189	(31.1)	182	(30.0)	221	(36.4)	15	(2.5)
**Female**								
(n = 430) (%)	149	(34.7)	139	(32.3)	122	(28.4)	20	(4.7)

p = 0.0198

The highest proportion of patients who were prescribed at least one off-label drug was children (85.2%) and neonates (83.3%) as inpatients (Table 2). Higher levels of unlicensed prescribing were observed in inpatient and outpatient infants (7.5% and 11.5% respectively). Differences in prescribing for inpatients and outpatients were significant (p = 0.0077 and 0.0004 respectively) but not for Emergency Department patients (p = 0.2514)

**Table 2:** Number and percentage of inpatients, outpatients and emergency department patients in various age groups prescribed no drugs, licensed, off-label or unlicensed drugs.

Patient Setting and Age	No Drugs		Licensed Drugs		Off-label Drugs		Unlicensed Drugs		p
	(n = 338)		(n = 321)		(n = 343)		(n = 35)		
**Inpatients** (n = 254)									0.0077
**0–27 d**									
(n = 12) (%)	1	(8.3)	1	(8.3)	10	(83.3)	0	(0.0)	
**28d—23 m**									
(n = 67) (%)	3	(4.5)	16	(23.9)	43	(64.2)	5	(7.5)	
**2–11 y**									
(n = 122) (%)	5	(4.1)	12	(9.8)	104	(85.2)	1	(0.8)	
**12–18 y**									
(n = 53) (%)	3	(5.7)	16	(30.2)	32	(60.4)	2	(3.8)	
**Outpatients** (n = 380)									0.0004
**0–27 d**									
(n = 2) (%)	0	(0.0)	1	(50.0)	1	(50.0)	0	(0.0)	
**28d—23 m**									
(n = 78) (%)	35	(44.9)	21	(26.9)	13	(16.7)	9	(11.5)	
**2–11 y**									
(n = 202) (%)	101	(50.0)	70	(34.7)	28	(13.9)	3	(1.5)	
**12–18 y**									
(n = 98) (%)	37	(37.8)	47	(48.0)	13	(13.3)	1	(1.0)	
**Emergency** (n = 403)									0.2514
**0–27 d**									
(n = 6) (%)	3	(50.0)	2	(33.3)	1	(16.7)	0	(0.0)	
**28d—23 m**									
(n = 133) (%)	53	(39.8)	42	(31.6)	32	(24.1)	6	(4.5)	
**2–11 y**									
(n = 200) (%)	74	(37.0)	62	(31.0)	56	(28.0)	8	(4.0)	
**12–18 y**									
(n = 64) (%)	23	(35.9)	31	(48.4)	10	(15.6)	0	(0.0)	

A total of 2654 drugs were prescribed to 699 patients. Of the 2654 prescriptions, 1905 (71.8%; 95% CI: 70.1% to 73%) were for licensed drugs, 681 (25.7%; 95% CI: 24.0% to 27.3%) for off-label drugs and 68 (2.6%; 95% CI: 2.0% to 3.2%) for unlicensed drugs. The number of drugs prescribed in each setting is shown in Table 3. More drugs were prescribed to inpatients (1494; 56.3%) than outpatients (502; 18.9%) or Emergency Department patients (658; 24.8%).

**Table 3:** Gender distribution showing the number of licensed, off-label and unlicensed drugs prescribed in each setting.

Setting(n = 2654)	Gender	Drugs Prescribed					
		Licensed		Off-label		Unlicensed	
		(n = 1905)		(n = 681)		(n = 68)	
**Inpatient**	**Male**(n = 1009) (%)	697	(69.1)	296	(29.3)	16	(1.6)
(n = 1494)	**Female**(n = 485) (%)	339	(69.9)	137	(28.2)	9	(1.9)
**Outpatient**	**Male**(n = 297) (%)	231	(77.8)	58	(19.5)	8	(2.7)
(n = 502)	**Female**(n = 205) (%)	167	(81.5)	26	(12.7)	12	(5.9)
**Emergency**	**Male**(n = 379) (%)	266	(70.2)	95	(25.1)	18	(4.7)
(n = 658)	**Female**(n = 279) (%)	205	(73.5)	69	(24.7)	5	(1.8)

The p-values (Chi-square test) to assess differences between genders in each setting were: p = 0.8572 (inpatient); p = 0.0361 (outpatient); p = 0.1182 (Emergency Department).

The number of drugs prescribed per patient ranged from zero to 21 (which included licensed, off-label and unlicensed drugs). Almost one-third of patients (32.6%) were prescribed no drugs while many received either one or two drugs (20.7% and 12.8% respectively). Seven inpatients were prescribed 21 drugs. Few inpatients were prescribed no drugs (4.7%). Frequently inpatients were prescribed three (11.0%), four (13.4%) or six (11.8%) drugs. The mean (SD, median, [quartiles]) number of drugs prescribed for inpatients was 5.88 (4.35, 5.00, [3#x2013;8]), outpatients 1.32 (1.82, 1.00, [0–2]) and Emergency Department 1.63 (2.5, 1.00, [0–2]). Many outpatients and Emergency Department patients were prescribed no drugs (38% and 46% respectively) or one drug (29% and 21% respectively). There were statistically significant differences in the proportions of drugs in each category prescribed to patients in each setting (p < 0.0001). The number of off-label drugs prescribed to each patient ranged from zero to 11 (inpatients), zero to five (outpatients) and zero to six (Emergency Department patients).

Table 4 shows that the main therapeutic classes of drugs were those for the nervous system (39.0%), alimentary tract and metabolism (15.4%) and anti-infectives for systemic use (15.1%). When all 2654 drugs were classified into their specific Anatomical Therapeutic Chemical classification categories (Table 4), the highest percentages of off-label prescribing was for drugs of the cardiovascular system (40.6%), respiratory system (39.4%), genitourinary system/ sex hormones (36.8%) and alimentary tract (34.1%). Drugs classified as off-label in the cardiovascular system included clonidine, hydralazine and nifedipine; the respiratory system included salbutamol and ipratropium; the genitourinary system/ sex hormones included sildenafil and solifenacin and the alimentary tract included ondansetron, metoclopramide and omeprazole.

**Table 4:** Frequency (percentage) of licensed, off-label and unlicensed drugs prescribed in each Anatomical Therapeutic Chemical classification (ATC) category.

Anatomical Therapeutic Chemical classification	Licensed		Off-label		Unlicensed		p-value
	(n = 1905)		(n = 681)		(n = 68)		
**Alimentary tract and metabolism** (n = 408) (15.4%)	258	(63.2)	139	(34.1)	11	(2.7)	0.0024
**Blood and blood forming organs** (n = 34) (1.3%)	28	(82.4)	3	(8.8)	3	(8.8)	0.1716
**Cardiovascular system**(n = 69) (2.6%)	37	(53.6)	28	(40.6)	4	(5.8)	0.0021
**Dermatologicals** (n = 111) (4.2%)	101	(91.0)	7	(6.3)	3	(2.7)	<0.0001
**Genito-urinary system and sex hormones** (n = 19) (0.7%)	10	(52.6)	7	(36.8)	2	(10.5)	0.0802
**Systemic hormonal preparations excluding sex hormones** (n = 154) (5.8%)	129	(83.8)	3	(1.9)	22	(14.3)	0.0016
**Anti-infectives for systemic use** (n = 400) (15.1%)	302	(75.5)	97	(24.3)	1	(0.2)	0.1253
**Antineoplastics and immunomodulating agents**(n = 42) (1.6%)	28	(66.7)	9	(21.4)	5	(11.9)	0.5008
**Musculo-skeletal system**(n = 73) (2.8%)	71	(97.3)	2	(2.7)	0	(0.0)	0.0002
**Nervous system**(n = 1034) (39.0%)	739	(71.5)	295	(28.5)	0	(0.0)	(reference)
**Antiparasitic products, insecticides and repellent**(n = 5) (0.2%)	4	(80.0)	1	(20.0)	0	(0.0)	0.6761
**Respiratory system**(n = 180) (6.8%)	106	(58.9)	71	(39.4)	3	(1.7)	0.0008
**Sensory organs**(n = 95) (3.6%)	63	(66.3)	19	(20.0)	13	(13.7)	0.2901
**Various** (n = 30) (1.1%)	29	(96.7)	0	(0.0)	1	(3.3)	0.0163

The p-values in the last column compare the proportion of licensed drugs within each ATC class against the proportion in the Nervous System ATC group (the largest group of drugs).

Within specific Anatomical Therapeutic Chemical classification categories, the highest percentage of unlicensed prescribing was for systemic hormonal preparations excluding sex hormones (14.3%), sensory organs (13.7%), and antineoplastics and immunomodulating agents (11.9%). Unlicensed drugs prescribed from each of these categories included dexamethasone (reformulated), Dilacaine (a Princess Margaret Hospital eye drop formulation) and azathioprine (reformulated).

The 10 most commonly prescribed drugs were paracetamol, ibuprofen, ondansetron, Painstop Day (120 mg paracetamol and 5 mg codeine phosphate per 5 mL), morphine, oxycodone, amoxicillin, dexamethasone, salbutamol and prednisolone. The 10 most frequently prescribed off-label drugs were ondansetron (13.8%), Painstop Day (10%), salbutamol (7.5%), oxycodone (7.2%), paracetamol (7.1%), midazolam (4.3%), fentanyl (3.1%), Timentin (ticarcillin with clavulanic acid) (2.8%), amoxicillin (2.6%) and flucloxacillin (2.6%).

The 10 most common off-label drugs prescribed to different age groups is shown in Table 5. Not surprisingly, the 10 most commonly prescribed drugs overall as well as off-label drugs in each age category were different with neonates prescribed more anti-infectives and adolescents prescribed more analgesics or other drugs classified for the nervous system.

**Table 5:** The ten most common off-label drugs prescribed to different age groups.

Drugs prescribed for 0–27 days			Drugs prescribed for 28 days—23 months			Drugs prescribed for 2–11 years			Drugs prescribed for 12–18 years		
(n = 94 drugs)			(n = 533 drugs)			(n = 1302 drugs)			(n = 725 drugs)		
**Drugs**	**Freq**	**%**	**Drugs**	**Freq**	**%**	**Drugs**	**Freq**	**%**	**Drugs**	**Freq**	**%**
Amoxicillin	9	(30.0)	Salbutamol	25	(15.2)	Painstop Day	60	(15.6)	Ondansetron	23	(23.0)
Gentamicin	4	(13.3)	Ondansetron	14	(8.5)	Ondansetron	57	(14.7)	Fentanyl	7	(7.0)
Aciclovir	3	(10.0)	Paracetamol	13	(7.9)	Oxycodone	40	(10.3)	Lorazepam	5	(5.0)
Omeprazole	3	(10.0)	Painstop Day	10	(6.1)	Paracetamol	34	(8.8)	Oxycodone	5	(5.0)
Augmentin Duo	2	(6.7)	Amoxicillin	7	(4.3)	Salbutamol	23	(5.9)	Temazepam	5	(5.0)
Dopamine	2	(6.7)	Flucloxacillin	7	(4.3)	Midazolam	16	(4.1)	Ticarcillin/ clavulanic acid	5	(5.0)
Midazolam	2	(6.7)	Midazolam	7	(4.3)	Fentanyl	13	(3.4)	Fluoxetine	4	(4.0)
Hydrocortisone	1	(3.3)	Omeprazole	7	(4.3)	Clonidine	11	(2.8)	Midazolam	4	(4.0)
Meropenem	1	(3.3)	Codeine	6	(3.7)	Flucloxacillin	11	(2.8)	Quetiapine	4	(4.0)
Metronidazole	1	(3.3)	Propofol	5	(3.1)	Metoclopramide	10	(2.6)	Tobramycin	4	(4.0)

Note: Augmentin Duo (amoxicillin/ clavulanic acid); Painstop Day (paracetamol/ codeine phosphate)

The majority of unlicensed drugs, which included cephazolin and Dilacaine eye drops, dexamethasone, azathioprine, propranolol, tinidazole, gonadorelin and tocilizumab, were reformulated due to a lack of availability of a commercial preparation. A few unlicensed and unregistered drugs, including tocilizumab, were imported since they were not available in Australia.

The most common reasons for off-label prescribing were due to age and dosage (43.2% and 47.4% respectively; Table 6). The percentage of off-label prescribing due to dosage would probably have been higher but once drugs were classified as off-label due to age (ie the first hierarchical category), then further classification of that set was not considered. This indicates that dosage was clearly the primary factor leading to off-label prescribing. The least common reason for off-label prescribing was indication.

**Table 6:** Reasons for off-label prescribing applying the hierarchical classification used.

Reason for off-label prescribing	Frequency	Percentage
	(n = 681)	
**Age**	294	43.2
**Indication**	29	4.3
**Route of administration**	35	5.1
**Dosage** (including inappropriate frequency)	323	47.4

Reasons for off-label prescribing varied with each Anatomical Therapeutic Chemical classification code with some therapeutic classes off-label all of the time for age, such as blood and blood forming products, dermatologicals, systemic hormonal preparations excluding sex hormones, drugs affecting the musculoskeletal system and antiparasitic products. Drugs affecting the respiratory system were more commonly off-label due to dosage (85.9%) rather than age (14.1%).

## Discussion

This is the first study of its kind involving a random selection of patient records to investigate the extent of off-label and unlicensed prescribing across all major patient settings in a paediatric hospital.

The proportion of off-label and unlicensed prescribing in this study (25.7% and 2.6% respectively) was higher than reported in a previous Princess Margaret Hospital study conducted more than a decade ago, in which 16.2% of drugs prescribed were off-label or unlicensed [22]. However, our findings were similar to a recent Australian inpatient study in Tasmania which reported 31.8% of drugs as off-label [23]. The results of the current study were different to those from a previous Australian study conducted in a neonatal intensive care unit more than 10 years ago, which reported 47% of drugs as off-label and 11% as unlicensed, with 80% of infants prescribed either an off-label or unlicensed drug [1]. The percentage of patients prescribed off-label or unlicensed drugs in the current study (36.5%) was similar to a previous Princess Margaret Hospital study but less than the 57.3% reported in a recent study in Tasmania [23].

The difference in the age of patients in O'Donnell's study [1] (gestational age of 22.7 to 41.4 weeks) and other Australian studies involving a wider age range of patients, such as Turner's study [22] (in which the age ranged from 49 days to 18 years), Ballard's study [23] (in which the age ranged from one day to 11 years) and the current study (age zero to 18.4 years), makes a direct comparison of rates inappropriate with other Australian studies. However, in the current study, when only inpatient neonates aged zero to 27 days were considered, despite some being slightly older than in O'Donnell's study, the extent of off-label prescribing was 83.3% which was considerably higher than the overall study result. Although the number of inpatients in this group was small (n = 12), the finding suggests that by comparing specific patients groups, including specific age groups, the extent of off-label prescribing reported is highly variable. In addition, limiting the age range to lower age groups would give a higher likelihood of an off-label classification, especially with respect to age. Neonates are treated on birth at another hospital in Western Australia unless they have been discharged in which case they would be expected to attend this hospital. This may explain the lower number in this sample.

The prevalence of off-label and unlicensed prescriptions in this study was lower than those reported in several overseas studies in the UK (55% and 10%) [5], Sweden, Italy, Germany, Netherlands (39% and 7%) [16], the Netherlands alone (44% and 28%) [7], Switzerland (25% and 24%) [10], France (63% and 10%) [6], Israel (59% and 16%) [24] and Malaysia (34% and 27%) [21]. However, the current findings were similar to a study in Germany which reported 26.3% off-label and 0.4% unlicensed prescribing [8]. The percentage of off-label prescribing in this study was also similar to the 30.5% reported by Hsien et al [9] in Germany. Other studies that reported lower off-label or unlicensed prescribing included studies in Israel (26% and 8%) [20], UK (18% and 7%) [3] and Croatia (12% and 13%) [13].

A contributing factor to the lower percentages of off-label and unlicensed prescribing reported in this study is that data were collected across all settings at Princess Margaret Hospital. To the best of our knowledge, no previous study has investigated off-label and unlicensed prescribing collectively in inpatients, outpatients and Emergency Department patients. Most studies, including those in Australia, have been conducted in a selected setting e.g. Emergency Department patients, medical or surgical wards or Intensive care units or outpatients and some have included only neonates or children of specific age groups.

Although the overall extent of off-label and unlicensed prescribing in this study was lower than many published studies, by selecting only specific groups of patients such as inpatients, it was found that off-label and unlicensed prescribing occurred to a greater extent in this group than in any of the other settings, with 77.3% of females and 77.8% of males prescribed one or more off-label or unlicensed drugs. The percentages were much lower for Emergency Department patients (29.2% of males and 26.4% of females) and outpatients (18.7% of males and 16.8% of females). The differences in the proportions of patients prescribed off-label and unlicensed drugs in the different settings was statistically significant (p < 0.0001). More off-label and unlicensed drugs were prescribed to inpatients (30.7%) compared to outpatients (20.7%) and Emergency Department patients (28.5%) (Table 3).

When specific age groups were analysed, off-label and unlicensed prescribing was more prevalent in neonates (n = 12; 60.0%) with 55% prescribed off-label drugs and 5% prescribed both off-label and unlicensed drugs. However, the sample size (20 patients) was small and the majority of neonates (60.0%) were inpatients. A high percentage of off-label and unlicensed drugs were also prescribed to infants (n = 108; 38.8%) and children (38.2%) (n = 200; 38.2%) compared to adolescents (n = 58; 27.0%). These differences were statistically significant overall (p = 0.0080), with the difference being entirely attributable to the lower rate in adolescents (infants vs adolescents p = 0.0059; children vs adolescents p = 0.0039, infants vs children p = 0.8503). This analysis confirms that children under 12 years of age are less likely to be included in the license specifications than adolescents (12–18 years of age).

Inpatients were prescribed between zero and 21 drugs, Emergency Department patients between zero and 16 drugs and outpatients between zero and 12 drugs. The median number of drugs prescribed was one drug for both Emergency Department patients and outpatients and five drugs for inpatients (overall study median was one drug). One reason for a higher median number of drugs for inpatients was that very few inpatients were prescribed no drugs (4.7%) compared to Emergency Department patients (38.0%) and outpatients (45.5%). This was similar to findings reported from an inpatient study in Tasmania, in which only 20 of 300 patients (6.7%) were prescribed no drugs [23]. The difference in the number of drugs prescribed in each setting was significant (p < 0.0001). There is variable reporting of patients who receive off-label or unlicensed drugs because some studies include patients that receive no drugs and others omit them from the calculation.

The overall median number of drugs prescribed in this study was lower than the median of four drugs reported by Turner [22]. However, Turner's study was conducted on inpatients and in this study, when only inpatients were considered, the median number of drugs (five) was slightly higher than reported by Turner (four). A contributing factor to these differences may be that Turner's study involved only two wards (a surgical and a medical ward) whereas in this study, patients were randomly selected from all inpatient wards at Princess Margaret Hospital. Furthermore, a recent study in Finland comparing prescribing trends in 2001 and 2011 also reported an increase in the median number of prescriptions after 10 years [12].

Overall, the most common Anatomical Therapeutic Chemical classification categories were the nervous system, alimentary tract and metabolism and anti-infectives for systemic use. Of the 10 most commonly prescribed drugs, half were analgesics/ antipyretics from the nervous system category (paracetamol, ibuprofen, Painstop Day, morphine and oxycodone). Several of these drugs, including paracetamol, ibuprofen, oxycodone and morphine, have been reported as commonly prescribed drugs in other studies [7, 10, 15, 17, 19, 23, 29]. In a previous study at Princess Margaret Hospital, Turner reported that paracetamol and morphine were commonly prescribed [22].

The most common reasons for off-label prescribing were dosage (47.4%) and age (43.2%). Many other researchers have also reported that the most common reason for off-label prescribing was dose and/ or dose frequency [5, 7, 15, 16, 18–20, 23, 30–32]. The percentage of off-label prescribing in this study due to dosage may have been higher but since the hierarchical classification by Hsien et al. [9] was used, once drugs were classified into a category above dosage (i.e. age, indication and route of administration) they were then not considered for another category.

Differences in study design and the variations in definition of the term "off-label" and "unlicensed" can make a direct comparison between studies inappropriate. For example, Santos et al. [18] reported that the most frequent reason for unlicensed medications was that ''safety and efficacy have not been established in children''. Most other researchers classified this situation as off-label rather than unlicensed [9] [22].

Approximately 35% of current prescribing to inpatient children and 28% for all categories of patients are prescribed drugs outside of the license requirements and therefore the efficacy and safety data associated with the use of these drugs in these populations is limited or non-existent It is notable that most of the drugs identified as off-label or unlicensed have been available for many years. The Therapeutics Goods Administration in Australia and regulatory authorities in many other countries require the sponsor to seek amendments to the Product Information data. This requirement therefore discourages any changes being submitted for changes to the registration details when off-label prescribing for such an indication enables patient access to the drug. A new approach could involve the establishment of an expert group to assess clinical trials reported for indications, dosages, age and route of administration. If data was assessed as efficacious and safe it should then be included in the Product Information. Some registration authorities are encouraging trials in paediatric groups for new drugs. However, currently long standing drugs are responsible for most of the current problematic situation. These studies could be distributed around the harmonised government agencies and potentially contribute to paediatric public health.

## Limitations of This Study

There were several limitations to this study. This study involved only a single centre which was a tertiary care centre. It is possible that in a tertiary care paediatric hospital (with a great proportion of complex cases) the rate of off-label prescribing is higher than in a community/ regional hospital. Further, there were few neonates as they were initially treated at another hospital. Therefore, this group is under-represented in the sample, so that conclusions regarding the small number of study patients in this group should be interpreted with caution.

Not all charts were available during the study period although every effort was made to access the charts. It was also expected that more patients would be prescribed psychotherapeutic drugs than found in the sample. A reason might be that some of these may have been child protection cases or they may have been on the ward at the time of data collection, in which case their medical record would not have been available for the study.

There were limitations with the hierarchical classification system because in a number of cases, if a hierarchical approach had not been used, the proportion of drugs classified as off-label due to indication, route of administration or dose would have been higher. The classification system may have led to a lower proportion of unlicensed drugs being reported. In some categories there are very low levels of unlicensed prescribing and hence an even larger sample would be necessary to provide more confidence in these estimates.

## Conclusion

The use of off-label and unlicensed prescribing was found to be high at Princess Margaret Hospital with an overall prevalence of 28.3%. The percentage of patients prescribed at least one off-label or unlicensed drug was 36.5% with a high percentage of off-label prescribing associated with nervous system drugs. The highest percentage of unlicensed prescribing was with systemic hormonal preparations excluding sex hormones.

This study provides the first data on the prevalence of off-label and unlicensed prescribing across all three main hospital settings.

These findings indicate that more than one-third of patients are being exposed to medicines for which prescribing information is not licensed in the Product Information. Children are considered a vulnerable group of patients and governments around the world need to be aware of the potential public health risk from these findings.

It is recommended that a government sponsored group of experts should rigorously consolidate and evaluate the quality of evidence for the prescribing of the common off-label drugs identified in this study. Where there are notable deficiencies studies are recommended to provide an adequate evidence-base for their prescribing so that they can be provided in a legally sanctioned manner or the licensed Product Information amended accordingly.
